# Exploiting Non-Markovianity for Quantum Control

**DOI:** 10.1038/srep12430

**Published:** 2015-07-22

**Authors:** Daniel M. Reich, Nadav Katz, Christiane P. Koch

**Affiliations:** 1 Theoretische Physik, Universität Kassel, Heinrich-Plett-Str. 40, D-34132 Kassel, Germany; 2 Racah Institute of Physics, The Hebrew University of Jerusalem, Jerusalem 91904, Israel

## Abstract

Quantum technology, exploiting entanglement and the wave nature of matter, relies on the ability to accurately control quantum systems. Quantum control is often compromised by the interaction of the system with its environment since this causes loss of amplitude and phase. However, when the dynamics of the open quantum system is non-Markovian, amplitude and phase flow not only from the system into the environment but also back. Interaction with the environment is then not necessarily detrimental. We show that the back-flow of amplitude and phase can be exploited to carry out quantum control tasks that could not be realized if the system was isolated. The control is facilitated by a few strongly coupled, sufficiently isolated environmental modes. Our paradigmatic example considers a weakly anharmonic ladder with resonant amplitude control only, restricting realizable operations to SO(N). The coupling to the environment, when harnessed with optimization techniques, allows for full SU(N) controllability.

Quantum control relies on the coherence of matter waves, employing external fields to steer the time evolution of a quantum system^1^,^2^. It holds the promise of utilizing entanglement and matter interference as cornerstones of future technologies. Typical control targets include the preparation of a state with certain properties, realization of a desired unitary operation, or generation of the maximum amount of entanglement. Accurate and reliable control solutions for such targets may be identified using optimal control techniques. As a prerequisite, the control target must be reachable. This question is addressed by controllability analysis.

For closed quantum systems, controllability is determined, besides the available resources such as power or bandwidth of the controls, by symmetries in the Hamiltonian^3^. Since the Hamiltonian generates the time evolution, its structure determines the directions in Hilbert space into which the dynamics can evolve and thus which states can be reached. For open quantum systems, the interaction with the environment leads to correlations which may induce memory effects in the system’s time evolution^4^. Such dynamics are termed non-Markovian. How the build-up of memory influences controllability is currently unknown, and control strategies for non-Markovian dynamics of open quantum systems remain largely uncharted territory.

Non-Markovian dynamics are generic for condensed phase settings encountered e.g. in light harvesting or solid-state devices. Non-Markovianity can be measured in terms of information flowing from the environment back into the system^5^, increase of correlations if the system is bi- or multipartite^6^, or re-expansion of the volume of accessible states^7^. While the debate on which measure is most useful for identifying non-Markovianity is on-going, see for example the discussion in Ref. 8, what is important in the context of quantum control is that each of these measures holds a promise for better control. Indeed, correlations between system and environment may improve fidelities of single qubit gates^9^, or assist one-photon phase control^10^. Cooperative effects of control and dissipation may allow for entropy export and thus cooling^11^; and harnessing non-Markovianity may enhance the efficiency of quantum information processing and communication^12^,^13^. For a system with limited controllability, one may conjecture that the coupling to the environment may provide the missing knobs, provided amplitude and phase flow back into the system.

Limited control over the system of interest is a generic feature in quantum engineering where controllability has to be balanced with sufficiently isolating the desired quantum features. A prototype model is a weakly anharmonic ladder, describing quantum systems as diverse as superconducting devices or phonons in an ion trap. With resonant amplitude control only, one can realize for such a ladder arbitrary operations in SO(N). Strikingly, for the special case of *N* = 4, the controls are known analytically since so-called Pythagorean couplings provide a generalization of Rabi cycling from two- to four-level systems^14^. Complete controllability requires, however, the ability to realize an arbitrary element of SU(N). Provided that one is able to implement any element of SO(N), an arbitrary element of SU(N) can be constructed based on the Cartan decomposition. It results in a decomposition of all unitaries *U*∈SU(N) into operations *k*_1_,*k*_2_ ∈ SO(N) and a diagonal, unitary matrix *A*^3^ such that *U* = *k*_1_*Ak*_2_. The task to achieve full unitary controllability on the *N*-level system therefore reduces to implementing an arbitrary diagonal unitary. This is the problem we address in the following, utilizing optimal control theory to properly exploit the coupling to the environment and ensure the desired phase alignment.

## Results

### Partioning the environment

Our approach starts from a complete microscopic description of system and environment. The environmental degrees of freedom are then modeled by an infinite set of oscillators or spins^15^. The system, by definition, is (fully or partially) controllable whereas no control can be exerted onto the environmental degrees of freedom. Nonetheless, the environment can be exploited to control the system. Key is a partitioning of the environment into a few strongly coupled, and potentially beneficial, modes and weakly coupled modes which result in dephasing and relaxation.

In our model, the strongly coupled modes are accounted for explicitly (“primary bath”) and taken to be two-level systems (TLS)^16^,^17^,^18^,^19^. Considering *n*_*p*_ such TLS, the Hamiltonian *H*_*QP*_, generating the evolution of system (“Q”) and primary bath (“P”), reads


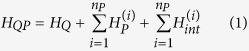


with *ħ* = 1. The system Hamiltonian *H*_*Q*_ describes an anharmonic ladder, *E*_*n*_ = *nω*_*Q*_ + *βn*(*n* + 1)/2, with base frequency *ω*_*Q*_ and anharmonicity *β* plus control by an external field, *u*(*t*), shifting the energy levels. For low anharmonicity, the shift is harmonic,


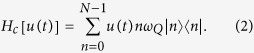


The *i*th primary bath-TLS is characterized by the splitting *ω*_*i*_, 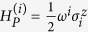, and couples transversally to the *N*-level system,


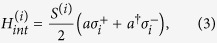


The coupling constant *S*^(*i*)^ corresponds to the system’s energy level splitting when on resonance with the *i*th TLS, and *a*^+^(*a*) denotes the creation (annihilation) operator of the *N*-level system.

Both system and primary bath-TLS interact weakly with the remaining environmental modes (“secondary bath”). Due to the weak coupling, these modes can be integrated over. As a result, system and primary bath modes are exposed to energy relaxation, caused by the near-resonant, weakly coupled secondary bath modes, as well as pure dephasing, due to the low-frequency secondary bath modes^15^. This is described by a Markovian master equation for the joint state of system and primary bath-TLS,


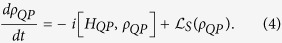


The state of the system alone, *ρ*_*Q*_, is obtained by integrating over the primary bath modes^16^,^17^. This is done *a posteriori*. Therefore, depending on the system’s coupling to the primary bath, the dynamics of *ρ*_*Q*_ may become non-Markovian (in contrast to the dynamics of *ρ*_*QP*_ which by construction is Markovian). Decay of the joint state of system and primary bath modes due to the secondary bath (“S”) is modeled by the Liouvillian 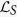 in the standard way^15^,


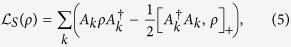


with 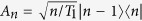 and 
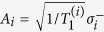
, affecting system level *n* and *i*th TLS, respectively. In order to limit the number of parameters and preserve clarity, we restrict our model to a *T*_1_-limited environment. In the supplemental material, we explicitly also scan dephasing times *T*_2_ and show that our main results and conclusions are unchanged when accounting for pure dephasing.

### Application to superconducting circuits

A good realization of our model is given by superconducting circuits. For example, resonant amplitude control using Pythagorean couplings has recently been demonstrated by high-frequency steering in a flux-biased Josephson phase circuit^20^. In this setup, frequency shifts as in Eq. (2) can be achieved by low-frequency steering of the bias flux^21^. This corresponds to neglecting terms that oscillate strongly on the timescale of *ω*_*Q*_. It is those terms that, for *N* = 4, yield SO(4) operations via the Pythagorean couplings^20^. Consequently the two control mechanisms do not interfere. For superconducting circuits, the primary bath-TLS correspond to strongly coupled dielectric defects 22,23,24. They can be characterized experimentally in terms of their splitting, coupling to the *N*-level system, *T*_1_ and *T*_2_^21^,^25^. Hamiltonian (3) neglects counter-rotating terms which is justified for couplings of the order of 50 MHz compared to bare system frequencies of about 5 GHz. Since these experiments are carried out in dilution refrigerators, all of the environment can be taken at *T* = 0 K.

### Controllability analysis

In the absence of the environment, the control Hamiltonian (2) does not allow for realizing arbitrary diagonal unitaries for the *N*-level system which would be required for complete SU(N) controllability. This is best analyzed in terms of the dynamic Lie algebra. It is obtained by generating all nested commutators of the summands in the system Hamiltonian^3^. The elements of the Lie algebra represent the Hilbert space directions along which the system can evolve. Therefore the rank of the dynamic Lie algebra needs to be equal to *N* − 1 for complete controllability in terms of diagonal unitaries^3^. For our model of an anharmonic ladder with controlled frequency shifts, the control and drift Hamiltonian commute, and evolution along only a single direction is possible. The scenario changes once the strongly coupled TLS of the primary bath come into play. In fact, a single transversal coupling term is sufficient to provide the remaining *N* − 2 Hilbert space directions, required for realizing an arbitrary diagonal unitary. This is due to *H*_*c*_ not commuting with 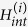. In more physical terms, 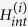 allows for the system wave function to be transferred to the TLS and back, after acquiring the desired non-local phases.

These considerations of controllability hold, however, only for unitary evolution. The environment also leads to irreversible loss of energy and phase. The only control strategy that is available for such Markovian dynamics is to beat decoherence (unless a protected region in Hilbert space exists in which the desired dynamics can be generated). It is thus crucial to carry out all operations as fast as possible. To this end we employ optimal control theory which allows for identifying controls that operate at the speed limit^26^. Our optimization target is *U*_1_ = diag(1, −1, 1, 1), and we quantify success in terms of the error, 1 − *F*_*average*_^27^. *U*_1_ is a particularly difficult unitary to implement, as exemplified by an error of over 40% in the absence of any strongly coupled TLS. While we discuss in the following only *U*_1_, we have verified that optimization towards diagonal unitaries with random phases yield very similar errors. For example, optimization for 20 random diagonal unitaries, using the same parameters for system and primary bath, yields errors that differ from that for *U*_1_ by less than a factor of 1.2. This suggests full SU(4)-controllability, once implementation of *U*_1_ is successful.

### Successful realization of an exemplary element of SU(4)

**Figure 1:**
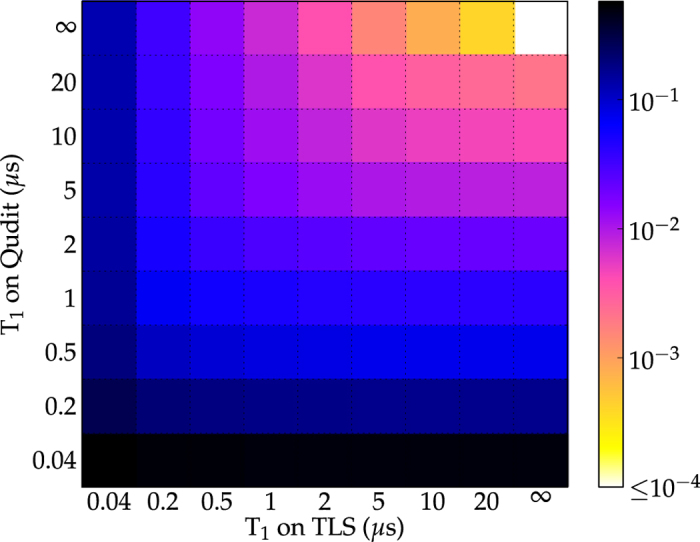
Realization of an examplary element of SU(4). Error after optimization for diag(1, −1, 1, 1) as a function of *T*_1_ times of qudit and TLS for an optimization time of *T* = 40 ns (anharmonicity *β* = 40 MHz, *ω*_*Q*_ − *ω*^(1)^ = 550 MHz, *S*^(1)^ = 60 MHz).

We first present results obtained with a single TLS in the primary bath and discuss the effect of additional TLS below. Figure 1 plots the error after optimization for *U*_1_ as a function of the *T*_1_ times of qudit and primary bath-TLS, demonstrating that errors below 1% can be reached even for *T*_1_ times of the order of a few microseconds. Due to increasing decoherence rate with increasing excitation, short *T*_1_ times of the qudit have a slightly more severe effect than short *T*_1_ times of the TLS.

**Figure 2:**
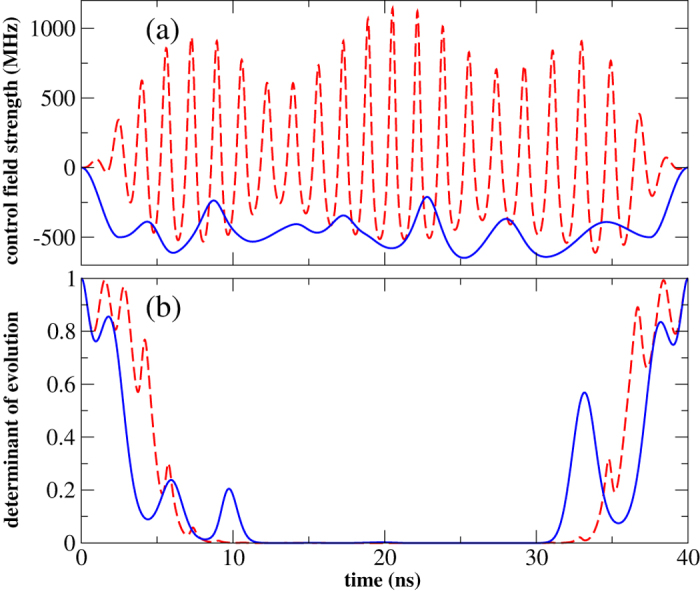
Optimized controls and non-Markovianity measure. (**a**): Optimized amplitudes with the control shown in blue following a fixed ramp of ±500 MHz over 2.5 ns at the beginning and end and the red dashed line obtained without imposing a ramp. (**b**): Liouville space determinant of the system evolution—increase of the determinant indicates non-Markovianity (parameters as in Fig. 1, 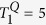 *μ*s, 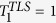 *μ*s).

A multitude of controls lead to the results shown in Fig. 1. Two examples of optimized controls, obtained using different constraints, are displayed in Fig. 2(a): The control can be restricted to low bandwidth by ramping it into and out of resonance at the beginning and end of the optimization time interval (blue solid line in Fig. 2(a)), whereas fast oscillating controls are obtained without imposing a ramp (red dashed line in Fig. 2(a)). The different controls all share the mechanism of moving the qudit close to resonance with the TLS, picking up a non-local phase due to the enhanced interaction, and moving the qudit back off resonance. This sequence is repeated several times in order to properly align all the phases in the four-level subspace. A visualization of the dynamics is provided as supplemental material. While both controls lead to similar errors, the ramped control is easier to implement experimentally and also fulfills the low-frequency approximation used to derive the control Hamiltonian (2). All further calculations therefore employ ramped controls.

Both solutions shown in Fig. 2(a) use the non-Markovianity of the time evolution as a core resource for control. This is seen in Fig. 2(b) which plots the determinant of the volume of reachable system states^7^. An increase of this quantity indicates a non-Markovian evolution of the qudit. Pronounced non-Markovianity is observed at the beginning and the end of the time interval in Fig. 2(b). The state space determinant is a comparatively weak non-Markovianity measure, that is, the evolution may also be non-Markovian at intermediate times. It will be interesting to assess how much non-Markovianity is required for control and over which extent of time. This is, however, beyond the scope of our present work which demonstrates the basic feasibility of exploiting non-Markovian dynamics for control.

### Dependence of control success on system and bath parameters

**Figure 3:**
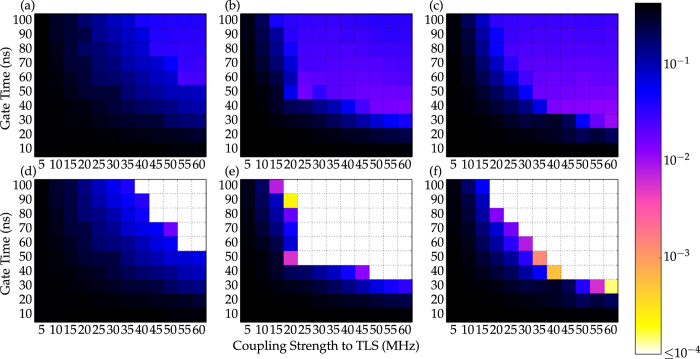
Dependence of control success on system anharmonicity. Error after optimization for diag(1,−1,1,1) for 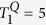 *μ*s, 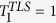 *μ*s (**a**–**c**) and anharmonicities of *β* = 0 MHz (**a**,**d**), 40 MHz (**b**,**e**), 150 MHz (**c**,**f**). For comparison, the error is also shown for infinite *T*_1_ of qudit and TLS (**d**–**f**).

Use of the environment as a resource is further illustrated in Fig. 3 which explores the dependence of the best possible error on qudit anharmonicity and coupling strength between qudit and TLS: For very small coupling no solution can be found and the error remains of the order one. On the other hand, one moderately coupled TLS in the primary bath is sufficient to yield good fidelities even for weak or zero anharmonicity. In the latter case (Fig. 3(a)), the desired diagonal unitaries can be realized if the operation time is sufficiently long. This can only be exploited for good *T*_1_ times, utilizing the level-dependent coupling strengths. The control problem becomes much easier for non-zero anharmonicity, with a subtle interplay between the requirements of resolving the qudit levels and sufficient interaction with all qudit levels. The latter corresponds to small anharmonicity (Fig. 3(b)) and subsequently allows good results even for weak coupling, whereas energy resolution is best for larger anharmonicity (Fig. 3(c)), which in turn allows for very short operation times. For fixed anharmonicity, one expects larger coupling strengths and longer gate times to allow for better fidelities. A few exceptions to this rule, which are observed in Fig. 3, can be attributed to the numerical nature of our controllability analysis. The observation that for a very weakly coupled TLS there exists no anharmonicity and no gate time that lead to even moderate fidelities is clear evidence that the primary bath is essential for the generation of arbitrary diagonal unitaries.

While the environment may provide interactions with the system that can be used as a resource for control, it can also have detrimental effects on the system. This is likely to happen when several strongly coupled modes affect the dynamics. Since the number, position and coupling strength of the TLS cannot be controlled in the preparation of actual devices, we analyze the presence of an additional TLS in our optimizations, cf. Table 1. If the TLS are not too close to each other, a suitable control can suppress the effect of the additional TLS even if it is strongly coupled and very noisy. On the other hand, and not surprisingly so, the stronger a closely lying second TLS is coupled to the qudit, the more difficult it is to maintain good fidelities. This is due to the fact that the gate time needs to be sufficiently long to resolve the energy difference between the two TLS. Adding more TLS to the primary bath does not change the picture shown in Table 1: In optimizations with as many as four strongly coupled primary bath TLS, the error is increased by less than a factor of 2 compared to the error for a single TLS if none of the additional TLS is close to the favourable one and less than a factor of 4 if a moderately lossy TLS is in its vicinity.

## Discussion

In summary, we have shown that non-Markovianity can be exploited for quantum control, enabling realization of all quantum operations in SU(4) where the system alone allows only for SO(4). Extension to SO(N) is straightforward: For *N* > 4, resonant amplitude-controls to realize arbitrary elements of SO(N) can be obtained numerically. As shown in the supplementary material, the required computational resources scale very moderately with *N*. The enhanced controllability results from an effective control over the system-bath coupling by moving the system into and out of resonance with a selected bath mode. Fast implementations of this control scheme can be obtained with optimal control theory such that the errors are solely *T*_1_-limited. Our model and results are directly applicable to superconducting phase and transmon circuits for which we predict, with reasonably simple controls, errors below one per cent for state of the art decoherence times.

More generally, our results provide a new perspective on open quantum systems—the environment can act as a resource for (almost) unitary quantum control. It can be exploited using optimal control theory as a tool to properly engineer the details of the dynamics. It requires one or a few environmental modes to be sufficiently isolated and sufficiently strongly coupled to the system. These conditions are met for a variety of solid-state devices other than superconducting circuits, for example NV centers in nanodiamonds or nanomechanical oscillators. In addition, on an abstract level, our work calls for a comprehensive investigation of controllability of open quantum systems, in order to gain a more rigorous understanding of when and how non-Markovianity is beneficial for quantum control.

## Methods

The equation of motion (4) was solved by calculating a polynomial approximation of the evolution operator, 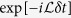 with 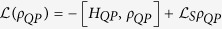, using Newton polynomials^28^. The time step *δt* was chosen sufficiently small to neglect time ordering.

To obtain a shape of the external control *u*(*t*) in Eq. (2) that realizes, as best possible, the desired unitary evolution, a recent variant of optimal control theory for unitary gates in open quantum systems^29^ was employed.

Non-Markovianity of the system evolution is assessed by computing the determinant of the volume of reachable system states^7^. While this measure for non-Markovianity is comparatively weak, it has, unlike all other non-Markovianity measures, the advantage of easy numerical evaluation. Its weakness compared to the non-Markovianity measures based on the divisibility of the dynamics^6^ or the non-monotonicity of the trace distance^5^ is of no concern for our purposes. That is, if a weak measure detects non-Markovianity, the dynamics clearly is non-Markovian also from the perspective of stronger measures.

## Additional Information

**How to cite this article**: Reich, D. M. *et al*. Exploiting Non-Markovianity for Quantum Control. *Sci. Rep*. **5**, 12430; doi: 10.1038/srep12430 (2015).

## Supplementary Material

Supplementary Information

## Figures and Tables

**Table 1:** Dependence of control success on primary bath parameters.

Δ^(2)^	*S*^(2)^	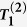	error
50 MHz	40 MHz	2000 ns	3.076 · 10^−2^
50 MHz	40 MHz	200 ns	4.052 · 10^−2^
50 MHz	40 MHz	40 ns	7.867 · 10^−2^
50 MHz	10 MHz	2000 ns	3.196 · 10^−2^
50 MHz	10 MHz	200 ns	3.564 · 10^−2^
50 MHz	10 MHz	40 ns	4.241 · 10^−2^
450 MHz	40 MHz	2000 ns	1.659 · 10^−2^
450 MHz	40 MHz	200 ns	1.652 · 10^−2^
450 MHz	40 MHz	40 ns	1.758 · 10^−2^
450 MHz	10 MHz	2000 ns	1.663 · 10^−2^
450 MHz	10 MHz	200 ns	1.674 · 10^−2^
450 MHz	10 MHz	40 ns	1.675 · 10^−2^

Error after optimization for diag(1, −1, 1, 1) with two primary bath TLS (parameters for qudit and first TLS as in Fig. 2, second TLS positioned Δ^(2)^ below *ω*^(1)^). For comparison, the error obtained for a single TLS is 1.652 · 10^−2^.
