# Supplementary material for: Exploiting Non-Markovianity for Quantum Control
Source: Sci Rep. 2015 Jul 22;5:12430. doi: 10.1038/srep12430 (PMC4510487; doi:10.1038/srep12430)
Supplement: Supplementary Information [file srep12430-s1.pdf]

Supplementary Material:
Exploiting Non-Markovianity for Quantum Control

Daniel M. Reich,[1] Nadav Katz,[2] and Christiane P. Koch[1]

[1]*Theoretische Physik, Universität Kassel, Heinrich-Plett-Str. 40, D-34132 Kassel, Germany*
[2]*Racah Institute of Physics, The Hebrew University of Jerusalem, Jerusalem 91904, Israel*

(Dated: April 29, 2015)

Capturing all detrimental effects by T_1 terms in the Lindblad master equation

In the following we provide simulation results which support our claim that the detrimental effect of pure dephasing can be accounted for by Markovian dynamics due to T_1 decay. To this end, we add to the Liouvillian, cf. Eq. (4) of the main paper, pure dephasing terms that are characterized by timescales T_2^* for both qudit and primary environment TLS. They read $A_n = \sqrt{2n^2/T_2^*}\,|n\rangle\langle n|$ for the qudit and $A_i = \sqrt{2/T_2^{*(i)}}\,|e_i\rangle\langle e_i|$ for the ith TLS where $|e_i\rangle$ refers to the energetically higher state. These dissipators cause the coherences to decay as a function of the square of the level difference instead of the linear relationship for energy relaxation. Low T_2^* times on the qudit have a more detrimental effect than low T_2^* for the TLS for the same value of T_2^*, due to the higher levels of the qudit.

Figure 1 presents the final fidelities obtained for optimizing in the presence of pure dephasing on top of T_1 decay. The decrease in fidelity with decreasing T_2^* times is very similar to the one observed for a T_1-limited environment in the main paper. The dynamics under the pulses optimized in the presence of T_2^* and T_1 is also very similar to that under the pulses optimized in the presence of T_1 alone. That is, the pulses move the qudit into and out of resonance with the two-level system, and it is only the alignment of the phases which is different for each individual pulse. This similarity is not surprising since, for the implementation of a unitary transformation, loss of phase and loss of population are equally bad; and only a short gate times can diminish their impact. The effect of pure dephasing on the control can therefore indeed by captured in terms of relaxation alone.

It is important, however, to estimate the increase of T_1 to properly account for the detrimental effect of pure dephasing, since in this case the coherences decay faster: At worst, the decoherence induced by a given T_2^* time is $2(N-1)$ times stronger than that due to an identical T_1 time, i.e., six times stronger for the qudit and twice as strong for the TLS. This factor arises for the loss of coherence between the highest and the lowest level of an N-level system. Taking these factors into account, one can explore the limitations on control due to Markovian effects with a single parameter, T_1, as has been done in the main paper.

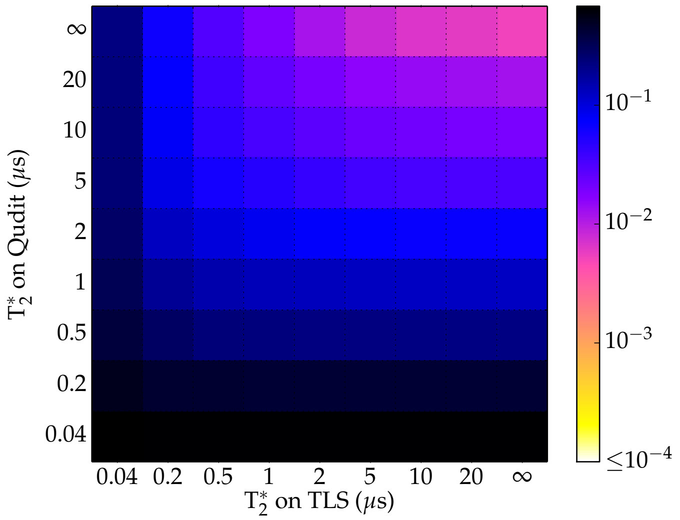

FIG. 1: Error after optimization for diag$(1,-1,1,1)$ as a function of the T_2^* times with T_1 fixed to $10\,\mu$s for both qudit and TLS (optimization time, $T = 40\,$ns, anharmonicity $\beta = 40\,$MHz, $\omega_Q - \omega^{(1)} = 550\,$MHz, $S^{(1)} = 60\,$MHz).

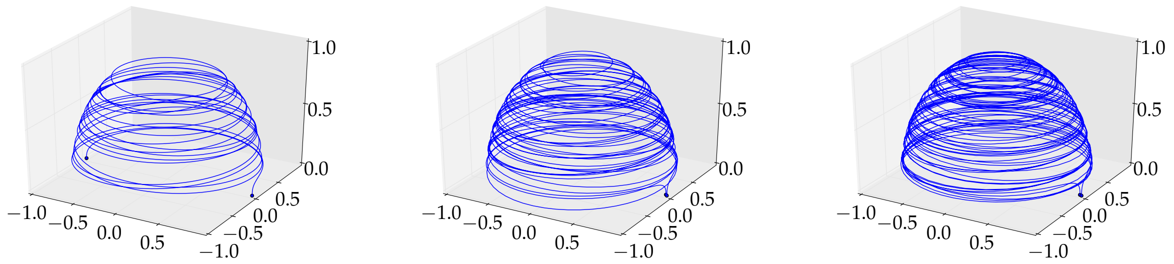

FIG. 2: Time evolution of system levels $n = 1, 2, 3$ (left to right) in the subspace spanned by $|n, g\rangle$ and $|n-1, e\rangle$ for the ramped control. Parameters as in Fig. 1 of the main paper, $T_1 = 5\,\mu$s (qudit), $T_1^{(1)} = 1\,\mu$s (TLS).

Visualization of the dynamics under the optimized controls

To visualize the optimized dynamics, we parametrize the joint state of system and primary bath two-level system (TLS) by $r(t)(e^{i\varphi(t)}\cos(\theta(t))\,|n, g\rangle + e^{i\chi(t)}\sin(\theta(t))\,|n-1, e\rangle)$ with g, e representing the TLS states. This makes use of the fact that, without loss due to finite T_1, any initial state $|n, g\rangle$ evolves in the subspace spanned by $|n, g\rangle$ and $|n-1, e\rangle$; and r, equal to the population in this subspace, accounts for population loss. The phase $\chi(t)$ is not relevant for our optimization problem and a spherical coordinate representation is obtained (with $\theta = 0$ corresponding to the xy-plane). Figure 2 displays the time evolution of each initial state $|n, g\rangle$, $n = 1, 2, 3$, under the optimized control (level 0 is coupled to neither control nor the rest of Hilbert space): Level 1 acquires a final phase of π, whereas levels 2 and 3 return to their initial position as desired. During the evolution, the radii become slightly smaller than one, indicating loss of population due to Markovian decay. At intermediate times, the points leave the xy-plane which indicates population transfer to the TLS. The different numbers of revolutions correspond to the different frequency shifts the individual levels acquire via the control. An animated version of Fig. 2 which illustrates synchronization of the dynamics of each of the levels is provided online.

Numerical optimization for anharmonic ladders with $N > 4$

In the following we provide simulation results which support the claim that our approach can easily be extended to N-level systems with $N > 4$. The main purpose of this section is to demonstrate feasibility of the required numerical calculations. The system parameters are chosen equivalently to the main paper for the $N = 4$ case, i.e., an initial detuning of $\omega_0 - E_{TLS} = 550\,$MHz, anharmonicity of $\beta = 40\,$MHz, and an on-resonance transversal coupling between TLS and qudit of 60 MHz. The ramping time is set to 2.5 ns, the ramping range to 500 MHz. The optimization time has been increased to 100 ns to account for the slightly more difficult optimisation problem. To be able to compare the numerical effort of the optimization as N is increased, we assume that any diagonal unitary can almost perfectly be reached, i.e., we set T_1 and T_2^* equal to 1 s for qudit and TLS. The dissipators are equivalent to those in the main text, adapted to the higher dimensionality.

We consider the following exemplary elements of SU(N) and SO(N) as optimization targets,

$$D = \begin{pmatrix} 1 & 0 & 0 & \cdots & 0 \\ 0 & -1 & 0 & \cdots & 0 \\ 0 & 0 & 1 & \cdots & 0 \\ \vdots & \vdots & \vdots & \ddots & \vdots \\ 0 & 0 & 0 & \cdots & 1 \end{pmatrix}, \qquad O = \begin{pmatrix} 0 & 0 & \cdots & 0 & 1 \\ 0 & 1 & \cdots & 0 & 0 \\ \vdots & \vdots & \ddots & \vdots & \vdots \\ 0 & 0 & \cdots & 1 & 0 \\ -1 & 0 & \cdots & 0 & 0 \end{pmatrix}. \tag{1}$$

D is the extension of U_1 in the main text to arbitrary N, whereas O corresponds to a flip between the lowest and highest level with all remaining levels left invariant. For the optimization of D, drift and control Hamiltonians are equivalent to those in the main text. In contrast, for the optimization of O, the drift Hamiltonian is transformed to the rotating frame in which all qudit levels are degenerate [1], i.e., effectively $\beta = 0\,$MHz. The control Hamiltonian

N	$\mathcal{I}$	$T_{\text{iteration}}^{\text{parallel}}$	$T_{\text{iteration}}^{\text{serial}}$	$T_{\text{iteration}}^{\text{serial}}(N)/T_{\text{iteration}}^{\text{serial}}(4)$
4	40	0.4 s	2 s	1.0
5	97	0.5 s	5 s	2.5
6	192	1 s	11 s	5.5
7	240	2 s	22 s	11.0
8	322	3 s	42 s	21.0
9	778	5 s	71 s	35.5
10	1094	7 s	116 s	58.0
4	4	1 s	9 s	1.0
5	2	3 s	24 s	2.7
6	8	5 s	61 s	6.8
7	8	9 s	135 s	15.0
8	14	16 s	279 s	31.0
9	8	30 s	512 s	56.9
10	23	51 s	906 s	100.7

TABLE I: Numerical effort required to find controls that realize the desired target (top: D, bottom: O) with a gate error below 1%: $\mathcal{I}$ denotes the number of iterations required to reach the 1% threshold, $T_{\text{iteration}}$ is the CPU time per iteration in parallel or serial mode. The scaling is emphasized by the last column which displays the CPU time per iteration (in serial mode) relative to that required for $N = 4$.

for realizing SO(N) operations is given by

$$H_c\left[\{u_k\left(t\right)\}\right] = \left[\sum_{k=1}^{N-1} iu_k\left(t\right)\left|k\right\rangle\left\langle k+1\right| - iu_k\left(t\right)\left|k+1\right\rangle\left\langle k\right|\right], \tag{2}$$

where all $u_k\left(t\right)$ are real. Equation (2) represents the generalization of the control Hamiltonian giving rise to Pythagorean control [1, 2] to $N > 4$. In the context of superconducting qubits, such a control Hamiltonian would be realized by high-frequency steering, in contrast to the low-frequency operation utilized in the control Hamiltonian of the main paper. As for the analytical solutions for $N = 4$, using Pythagorean couplings [1], we assume independent controls on the individual transitions, such that a total of $N - 1$ controls are optimized. For SO(N) control, the qudit is operated far away from any strongly coupled two-level system. All other parameters take the same value as for the diagonal unitary, except for the fact that the controls are not ramped.

The numerical effort to carry out optimization for D and O is listed in Table I as a function of N. All optimizations were performed on a multi-core processor with 24 cores. $\mathcal{I}$ refers to the number of iterations required to achieve a gate error below 1%. In all optimizations, the guess pulses, optimization weights and step sizes were chosen identically, independent of N. While adapting these optimization parameters may lead to a better performance, the results listed in Table I already demonstrate the feasibility of these calculations and the moderate increase of the numerical effort with N. The results in Table I are rationalized as follows: The average time per iteration increases due to the larger Hilbert space dimension, i.e., it reflects directly the cost for applying the Liouvillian to the state when evaluating the polynomial propagator, see Methods in the main text for more details. This cost scales as N^m where $2 < m < 3$ (it scales as N^3 in general but this scaling can be reduced by taking advantage of the sparsity of the Liouvillian). The average time per iteration differs for optimization of D and O due to the different system Hamiltonians (it is the spectral range of the Hamiltonian that determines the numerical cost of one time step). For optimization of O, the number of iterations required to reach the error threshold remains very moderate and increases, at least very roughly, about linearly with N. This indicates only a minor growth of the numerical complexity of the optimization. The increase in the number of iterations required to reach the error threshold is significantly larger for optimization of D but the calculations remain very feasible all the way up to $N = 10$ even in serial mode. Similarly to the different CPU times per iteration, this difference is also explained by the different system Hamiltonians. For optimization of O, the anharmonicity has been eliminated from the picture, that is, the difficulty in properly accounting for the anharmonicity is hidden in the assumed capability to produce $N - 1$ independent controls (note that this assumption had also been made for the analytical control solutions employed for $N = 4$ in Ref. [1]). In contrast, the anharmonicity is present in the optimizations for D. This leads to a quantitatively different behaviour since the additional levels have to be simultaneously left invariant by the optimized control field when implementing O. Therefore the complexity

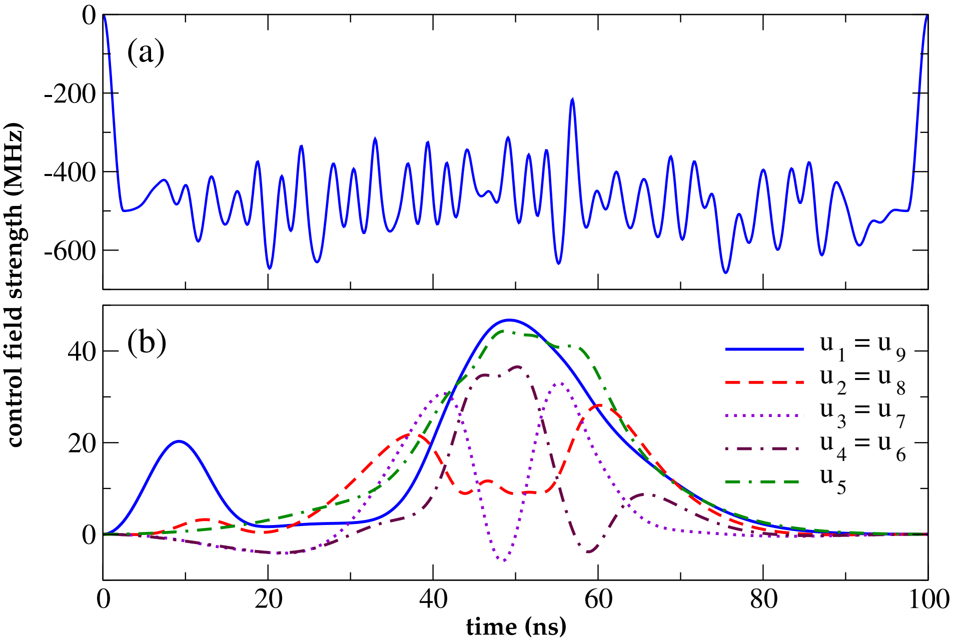

FIG. 3: Optimized controls to realize D (a) and O (b) for $N = 10$.

of the optimization problem increases with N. Nevertheless, the overall effort only scales algebraically (with a low power) with N.

Finally, the optimized controls obtained for $N = 10$ are presented in Fig. 3. Optimization for D yields a result very similar to the one obtained for $N = 4$ in the main text, with the only difference that more oscillations of the control are required. This is due to the requirement of proper phase alignment for ten compared to four levels (see also Fig. 2 for an illustration of the phase alignment problem). The controls obtained for optimizing O are characterized by an even simpler shape. The symmetry in the control amplitudes ($u_1 = u_9$ etc.) is due to the choice of the rotating frame. Note that while the numerically obtained control amplitudes are identical for u_i and u_{N-i}, the pulses in an experiment will differ due to the different transition frequencies between the levels which were absorbed in the u_k, cf. Eq. (2).

To summarize, we have demonstrated here the feasibility to obtain resonant amplitude controls to realize arbitrary elements of SO(N) numerically for $N \geq 4$. This substantiates our claim that extension of our control approach to $N > 4$ is straightforward.

[1] Svetitsky, E., Suchowski, H., Resh, R., Shalibo, Y., Martinis, J. M. & Katz, N. Hidden Two-Qubit Dynamics of a Four-Level Josephson Circuit. *Nature Commun.* **5**, 5617 (2014).

[2] Suchowski, H., Silberberg, Y. & Uskov, D. B. Pythagorean coupling: Complete population transfer in a four-state system. *Phys. Rev. A* **84**, 013414 (2011).
